# Reduced field-of-view DWI‑derived clinical–radiomics model for the prediction of stage in cervical cancer

**DOI:** 10.1186/s13244-022-01346-w

**Published:** 2023-01-26

**Authors:** Qiuhan Huang, Baodi Deng, Yanchun Wang, Yaqi Shen, Xuemei Hu, Cui Feng, Zhen Li

**Affiliations:** Department of Radiology, Tongji Hospital, Tongji Medical College, Huazhong University of Science and Technology, 1095 Jiefang Avenue, Qiaokou District, Wuhan, 430030 China

**Keywords:** Cervical cancer, Diffusion-weighted imaging, Tumor staging, Radiomics

## Abstract

**Background:**

Pretreatment prediction of stage in patients with cervical cancer (CC) is vital for tailoring treatment strategy. This study aimed to explore the feasibility of a model combining reduced field-of-view (rFOV) diffusion-weighted imaging (DWI)-derived radiomics with clinical features in staging CC.

**Methods:**

Patients with pathologically proven CC were enrolled in this retrospective study. The rFOV DWI with *b* values of 0 and 800 s/mm^2^ was acquired and the clinical characteristics of each patient were collected. Radiomics features were extracted from the apparent diffusion coefficient maps and key features were selected subsequently. A clinical–radiomics model combining radiomics with clinical features was constructed. The receiver operating characteristic curve was introduced to evaluate the predictive efficacy of the model, followed by comparisons with the MR-based subjective stage assessment (radiological model).

**Results:**

Ninety-four patients were analyzed and divided into training (n = 61) and testing (n = 33) cohorts. In the training cohort, the area under the curve (AUC) of clinical–radiomics model (AUC = 0.877) for staging CC was similar to that of radiomics model (AUC = 0.867), but significantly higher than that of clinical model (AUC = 0.673). In the testing cohort, the clinical–radiomics model yielded the highest predictive performance (AUC = 0.887) of staging CC, even without a statistically significant difference when compared with the clinical model (AUC = 0.793), radiomics model (AUC = 0.846), or radiological model (AUC = 0.823).

**Conclusions:**

The rFOV DWI-derived clinical–radiomics model has the potential for staging CC, thereby facilitating clinical decision-making.

**Supplementary Information:**

The online version contains supplementary material available at 10.1186/s13244-022-01346-w.

## Key points

- Radiomics features extracted from sagittal rFOV DWI were valuable in staging CC.
- Clinical factors including age and menopausal status were important predictors in staging CC.
- The clinical–radiomics model combining rFOV DWI-derived radiomics with clinical features is of great potential for staging CC, outperforming the radiological model.

**Supplementary Information:**

The online version contains supplementary material available at 10.1186/s13244-022-01346-w.

## Introduction

Cervical cancer (CC) ranks as the fourth most frequently diagnosed cancer and the fourth leading cause of cancer-specific death worldwide [1]. The treatment options for patients with CC mainly depend on its stages at initial diagnosis [2]. Patients with early-stage CC frequently undergo radical hysterectomy, whereas those with locally advanced CC are generally treated with concurrent chemo-radiotherapy [3]. Hence, accurate prediction of the stage in patients with CC is crucial for developing appropriate treatment strategies, and sparing patients with locally advanced CC from unnecessary surgery.

The revision of the 2018 International Federation of Gynecology and Obstetrics (FIGO) staging system acknowledges the central role of MRI in assessing the local extent of CC [4, 5]. With the advantages of tumor-to-normal tissue contrast, diffusion-weighted imaging (DWI) has been increasingly used to characterize CC [6]. The reduced field-of-view (rFOV) DWI, an MRI technique that enables minimizing the off-resonance induced artifacts by reducing the FOV in the phase-encoding direction, has been reported to considerably improve the conspicuity of CC [7, 8]. While the application of MRI in CC staging is still highly dependent on the subjective evaluation of radiologists [9], with poor stability and reproducibility. It is supposed that quantitative parameters may provide additional benefits. The apparent diffusion coefficient (ADC), which is derived from diffusion-weighted image, has also been successfully introduced to quantitatively characterize CC [10]. Its routine clinical use, nevertheless, has been hampered by the considerable overlap of ADC values among different tumor stages.

During the past years, radiomics has gained increasing interest in characterizing cancers [11]. It can extract additional quantitative data related to tissue microstructure from images, using automated and high-throughput extraction of data characterization algorithms to extract quantitative imaging features from a large number of medical images, deeply mining image information to improve decision support in oncology at low cost and noninvasively [12]. As radiomics may identify unique MRI features that reflect the underlying pathophysiology, it is expected to break through the limitation of the application of traditional imaging in early diagnosis, efficacy evaluation, and prognosis prediction of tumors [13]. At present, radiomics has been applied in many diseases with good performance, and its application in CC is gradually increasing [14].

So far, no study has been reported to evaluate the application of radiomics based on the rFOV DWI. Therefore, in this study, we aimed to explore the feasibility of the model combining rFOV DWI-derived radiomics with clinical features in predicting the stage of CC.

## Materials and methods

### Patients

**Fig. 1:**
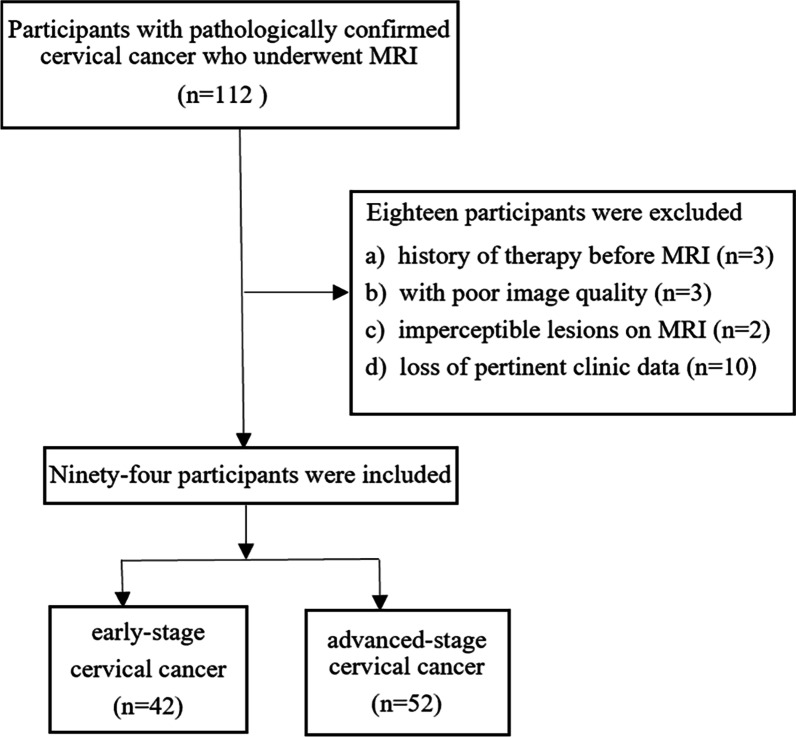
The flowchart of patient selection

This retrospective analysis was approved by the Ethics Committee of Tongji Hospital, and the requirement for informed consent was waived. One hundred and twelve patients with histopathologically confirmed CC were enrolled between March 2014 and August 2021. Inclusion criteria included: (a) patients with pathologically confirmed cervical cancer; (b) patients who underwent pretreatment MRI on a 3.0 T scanner. The exclusion criteria were as follows: (a) surgery or chemo-radiotherapy performed prior to MRI (*n* = 3); (b) inadequate image quality due to excessive artifacts (*n* = 3); (c) lesions with diameter less than 1 cm (*n* = 2); (d) incomplete clinic-pathological data (*n* = 10). The flowchart of patient selection is shown in Fig. 1.

Clinical characteristics of the enrolled patients were acquired from the electrical medical record system, including the information of age (years), menarche age (years), menopausal status (yes/no), number of gestation (times), number of production (times), and number of abortion (times) [15].

### MRI examination protocols

All participants underwent MR examinations on a 3.0 T MR scanner (High discovery 750, GE Medical Healthcare, Milwaukee, Wisconsin) in the supine position with a 32-channel torso phased-array coil. All recruited patients underwent axial T1-weighted imaging, T2WI, and rFOV DWI in our study. The scanning parameters of the T1WI, T2WI, and rFOV DWI are presented in Table 1.

**Table 1:** The acquisition parameters of each imaging sequence

	T1WI	T2WI	rFOV DWI
TR/TE (ms)	360/7.7	5755/136.9	3000/55.3
FOV (mm)	340 × 340	340 × 340	240 × 100
Matrix	256 × 256	320 × 256	128 × 96
Slice Thickness/gap (mm)	4/1	4/1	4/1
*b* values (s/mm^2^)	/	/	0, 800
Number of excitations	2	2	8
Band width (KHZ)	50	62.5	250
Orientation	Axial	Axial/coronal oblique/sagittal	Sagittal
Acquisition time (min)	1.5	2.5	2.5

*TR/TE* Repetition time/echo, *FOV* Field of view, *rFOV* Reduced FOV

### Image analysis

ADC maps were reconstructed by employing the mono-exponential model to fit to the sagittal rFOV DWI images with two *b* values (*b* = 0 and 800 s/mm^2^) using the following equation:

$$\mathrm{ADC}=\left[{\mathrm{lnS}}_{0}/\mathrm{lnS}\left(\left.b\right)\right.\right]/b$$

where *S*_*0*_ represents the signal intensity when *b* = 0 s/mm^2^; $\mathrm{S}\left(\left.b\right)\right.$ represents the signal intensity at a given *b* value.

The size of tumors was obtained based on the T2WI sequence by measuring the maximum diameter on the axial or coronal or sagittal images.

The tumor staging was performed by two radiologists independently based on the T2-weighted and diffusion-weighted images according to the FIGO 2018 staging system, without the knowledge of the clinical and histopathologic findings.

### Tumor segmentation and feature extraction

**Fig. 2:**
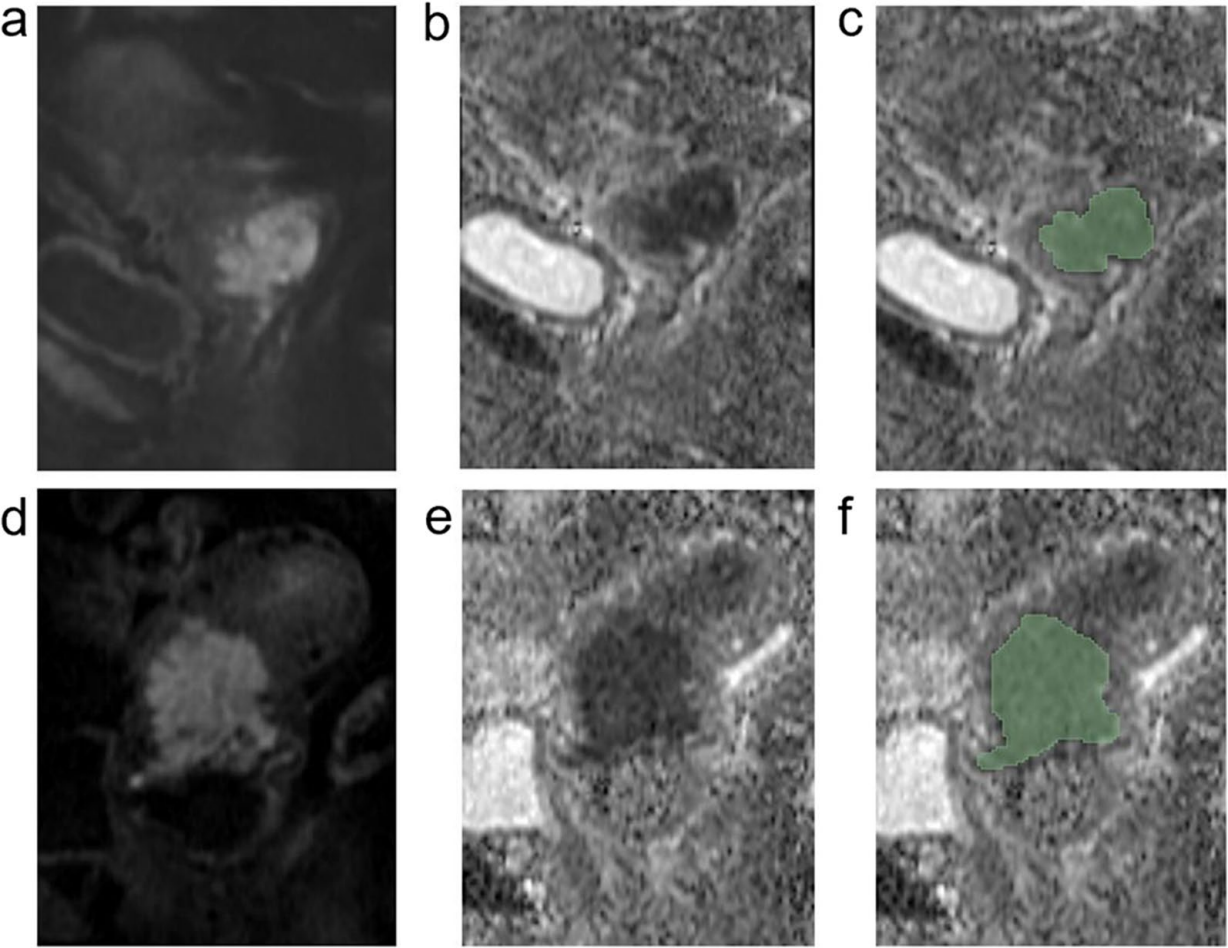
A patient with FIGO stage IB cervical cancer *(upper row)* and a patient with FIGO stage IV cervical cancer *(lower row)*. The high signal intensity on sagittal rFOV DWI with *b* value of 800 s/mm^2^ (**a** and **d**) and the low signal intensity on the corresponding ADC maps (**b** and **e**) illustrate the areas of tumors. The green areas on the ADC maps (**c** and **f**) represent the tumor regions of interest

The 3D Slicer software (version 4.13.0; www.slicer.org) was used for manual segmentation. The volumes of interest (VOIs) covering the whole tumor with low signal intensity were delineated along the tumor border layer by layer on the ADC maps of all the patients by a radiologist (C.F., with 11 years of pelvic MRI diagnosis experience), the corresponding T2WI and DWI images were also used for defining the anatomic structures and determining the tumor boundaries (Fig. 2). To analyze the inter-observer consistency, 30 patients were randomly selected and the VOIs of the tumors were delineated again by another radiologist (B.D.D., with 5 years of pelvic MRI diagnosis experience).

The 3D radiomics feature extraction was implemented by using MATLAB (2021b; https://www.mathworks.cn). Specific details of feature extraction are described in Additional file 1: Table S1.

### Radiomics feature selection and signature construction

The intra-class correlation coefficient (ICC) was conducted to assess the consistency between the two observers for each extracted feature. Only the features with good consistency (ICC > 0.8) were selected for further analysis. Then, features with statistically significant differences between the early- and advanced-stage CC were further selected by performing a *t* test, and a significance level of *p* < 0.1 was set.

The least absolute shrinkage and selection operator (LASSO) logistic regression algorithm with a ten-fold cross-validation was adopted for further dimension reduction and signature building. Nonzero coefficient features were selected and their linear combination was calculated to generate radiomics signatures. Radiomics score (Rad-score) was acquired for each patient with the following formula:

$$\mathrm{Rad}-\mathrm{score}=\mathrm{intercept}+{\beta }_{i}\times {X}_{i}$$

where *β* represents coefficient, *X* represents feature, and *i* represents an ordinal number.

### Construction and comparison of clinical, radiomics, radiological, and clinical–radiomics models

Four different models including the clinical (clinical predictors), radiomics (rad-score), radiological (subjective staging data), and clinical–radiomics models were constructed, respectively. The clinical–radiomics model combining clinical predictors and rad-score was built and then a clinical–radiomics nomogram was created by using logistic regression analysis in the training group. The receiver operating characteristic (ROC) curve was used to evaluate the discrimination performance of these four established models. In the training and testing cohorts, the area under the curve (AUC), sensitivity, and specificity of the models were obtained. At last, the decision curve analysis (DCA) was performed to quantify the net benefit at different threshold probabilities to determine the predictive value of the clinical–radiomics model.

### Statistical analysis

The statistical analysis was performed by using the SPSS (version 23.0, www.ibm.com/software/analytics/spss) and R software (version 3.6.1, www.R-project.org). The inter-observer reliability of the categorical variables was accessed by calculating the weighted kappa coefficient. The Kolmogorov–Smirnov test was used to assess the normality of all continuous variables. The *t* test or Mann–Whitney U test was conducted to assess the differences in age, menarche age, and tumor size between the training and testing cohorts. The Chi-squared test was used to evaluate the differences in the categorical variables such as histological classification, menopausal status, number of gestation, number of production, number of abortion, and FIGO stage between the training and testing cohorts. Univariate analysis (*t* test or rank sum test for continuous variables and Chi-square test for categorical variables) was used to select the significant predictive factors in discriminating early from advanced-stage CC in the training set. The following R packages were used: The “glmnet” was used to perform the LASSO logistic regression model, the “rms” package was used for multivariate binary logistic regression, nomogram construction, and the “pROC” package was used to construct the ROC curve. The AUCs between different models were compared by using the DeLong test. In all tests, a two-sided *p* value < 0.05 was described as statistically significant.

## Results

### Clinical characteristics

The characteristics of all patients are shown in Table 2. In this study, 18 patients were excluded based on the exclusion criteria, and a total of 94 patients with pathologically proven CC were ultimately enrolled. The patients were further dichotomized between the early stage (FIGO stage IB-IIA, *n* = 42) and the advanced stage (FIGO stage IIB-IV, *n* = 52) according to the FIGO 2018 staging system. Among the 94 patients, 61 patients (mean age, 52 ± 9 years) were divided into the training cohort and 33 patients (mean age, 53 ± 11 years) into the testing cohort. Between the training and testing cohorts, there was no significant difference in all clinical characteristics (all *p* > 0.05). In the training set, the univariate analysis revealed that age (*p* = 0.013) and menopausal status (*p* = 0.030) may be significant impacts in differentiating early from advanced-stage CC, as presented in Table 3.

**Table 2:** Clinical characteristics of the patients in the training and testing sets

Characteristics	Training set (n = 61)	Testing set (n = 33)	*p* value
Age (years)*	52 ± 9 (29–71)	53 ± 11 (29–74)	0.720
Menarche age (years)*	14 ± 2 (10–20)	14 ± 1 (13–17)	0.425
Tumor size (mm)*	53 ± 21 (24–120)	52 ± 13 (35–79)	0.848
Histological type			0.708
SCC	55 (90)	31 (94)	
ACA	6 (10)	2 (6)	
Menopause			0.730
Yes	31 (51)	15 (45)	
No	30 (49)	18 (55)	
Number of gestation			0.226
≤ 3	32 (52)	13 (39)	
> 3	29 (48)	20 (61)	
Number of production			0.474
≤ 3	50 (82)	25 (76)	
> 3	11 (18)	8 (24)	
Number of abortion			0.206
≤ 3	54 (89)	26 (79)	
> 3	7 (11)	7 (21)	
FIGO stage			0.215
IB	16 (26)	6 (18)	
IIA	12 (20)	8 (24)	
IIB	13 (21)	6 (18)	
III	15 (25)	5 (16)	
IV	5 (8)	8 (24)	

Numbers in parentheses are percentages except where otherwise indicated

*SCC* Squamous cell carcinoma, *ACA* Adenocarcinoma

*Numbers are means ± standard deviations, with ranges in parentheses

**Table 3:** Comparison of clinical data between patients with early and advanced cervical cancer in the training and testing sets

Characteristics	Training set			Testing set	
	IB-IIA (n = 28)	IIB-IV (n = 33)	*p* value	IB-IIA (n = 14)	IIB-IV (n = 19)
Age (mean ± SD, years)	49 ± 9	55 ± 9	0.013*	48 ± 9	57 ± 11
Menarche age, (mean ± SD, years)	14 ± 2	14 ± 2	0.294*	14 ± 1	14 ± 1
Menopause			0.030		
Yes	10	21		3	15
No	18	12		11	4
Number of gestation			0.873		
≤ 3	15	17		5	8
> 3	13	16		9	11
Number of production			0.201		
≤ 3	25	25		12	13
> 3	3	8		2	6
Number of abortion			0.693		
≤ 3	24	30		10	16
> 3	4	3		4	3

*SD* Standard deviation

* indicates *t* test, others are Chi-square test

### Feature selection and radiomics signature construction

**Fig. 3:**
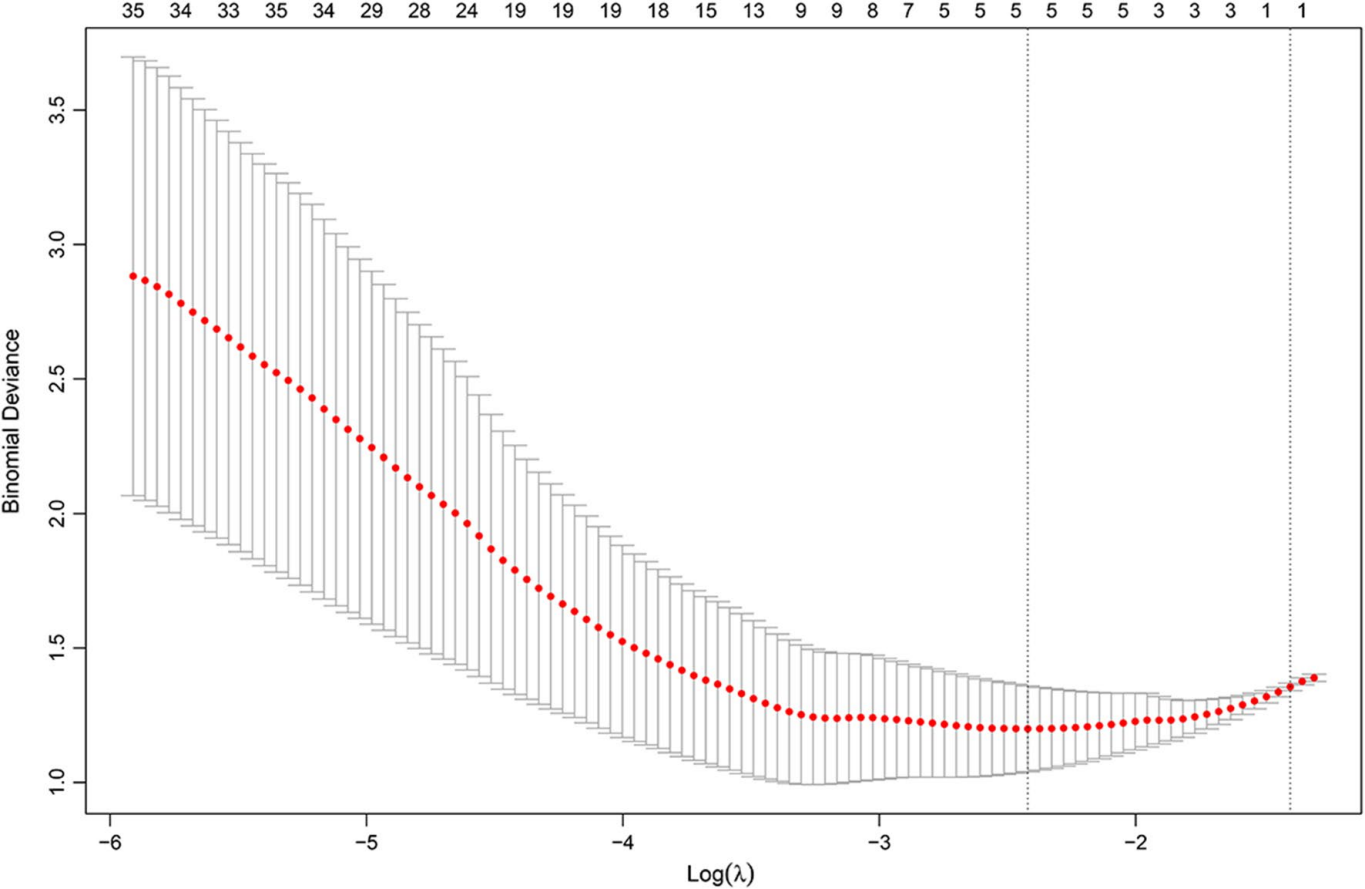
The radiomics feature selection using the LASSO regression analysis and the ten-fold cross-validation**.** The abscissa corresponding to the lowest point of the model deviation indicates the optimal penalization coefficient lambda (λ = 0.089)

A total of 851 radiomics features were extracted based on the sagittal rFOV DWI in this study, including the shape- and size-based features, first-order statistics features, textural features, and wavelet features. Then 320 radiomics features with good consistency (ICC > 0.8) and statistically significant differences between the early and advanced-stage groups were selected for further analysis. Through the LASSO algorithm using ten-fold cross-validation, five features (including one textural feature and four wavelet features) were finally chosen to build the radiomics signature, and the rad-score was constructed according to their corresponding coefficients. The process of LASSO analysis is shown in Fig. 3. The coefficients of the radiomics features are shown in Table 4. The detailed formula of the rad-score is presented in Additional file 1: Appendix 1.

**Table 4:** Coefficients for the calculation of the selected radiomics features

Variables	Coefficients
Intercept	− 25.62904339
Original_ NGTDM_ Contrast	− 1.883115529
Wavelet-LLH_GLCM_IMC2	− 0.982822030
Wavelet-LHH_GLCM_IDMN	32.062049922
Wavelet-LLL_ first-order _Robust Mean Absolute Deviation	− 0.001128832
Wavelet-LLL_ GLCM_ Difference Entropy	− 0.812853027

*NGTDM* Neighboring gray tone difference matrix, *GLCM* Gray-level co-occurrence matrix, *IMC2* Informational measure of correlation2, *IDMN* Inverse difference moment normalized

For inter-observer reliability of MRI-based FIGO stage evaluation between the two radiologists, a weighted kappa coefficient of 0.813 (95% CI: 0.725, 0.901) was achieved, indicating good consistency.

### Development, performance, and clinical use of prediction models

**Fig. 4:**
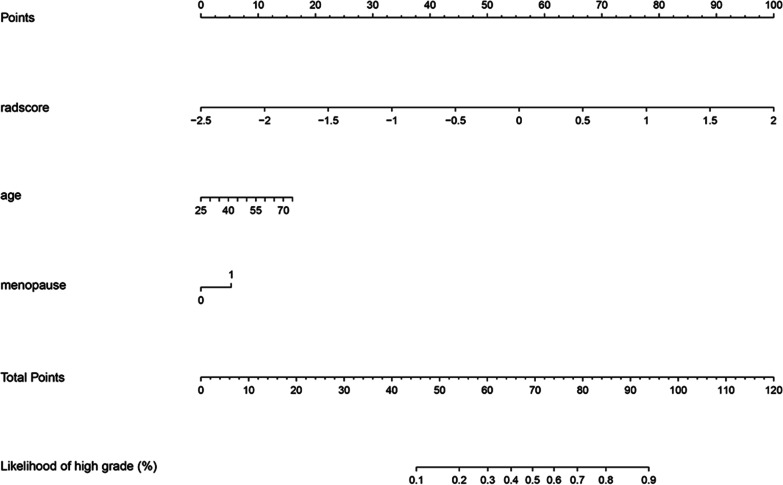
The nomogram based on the rad-scores, age, and menopausal status

**Fig. 5:**
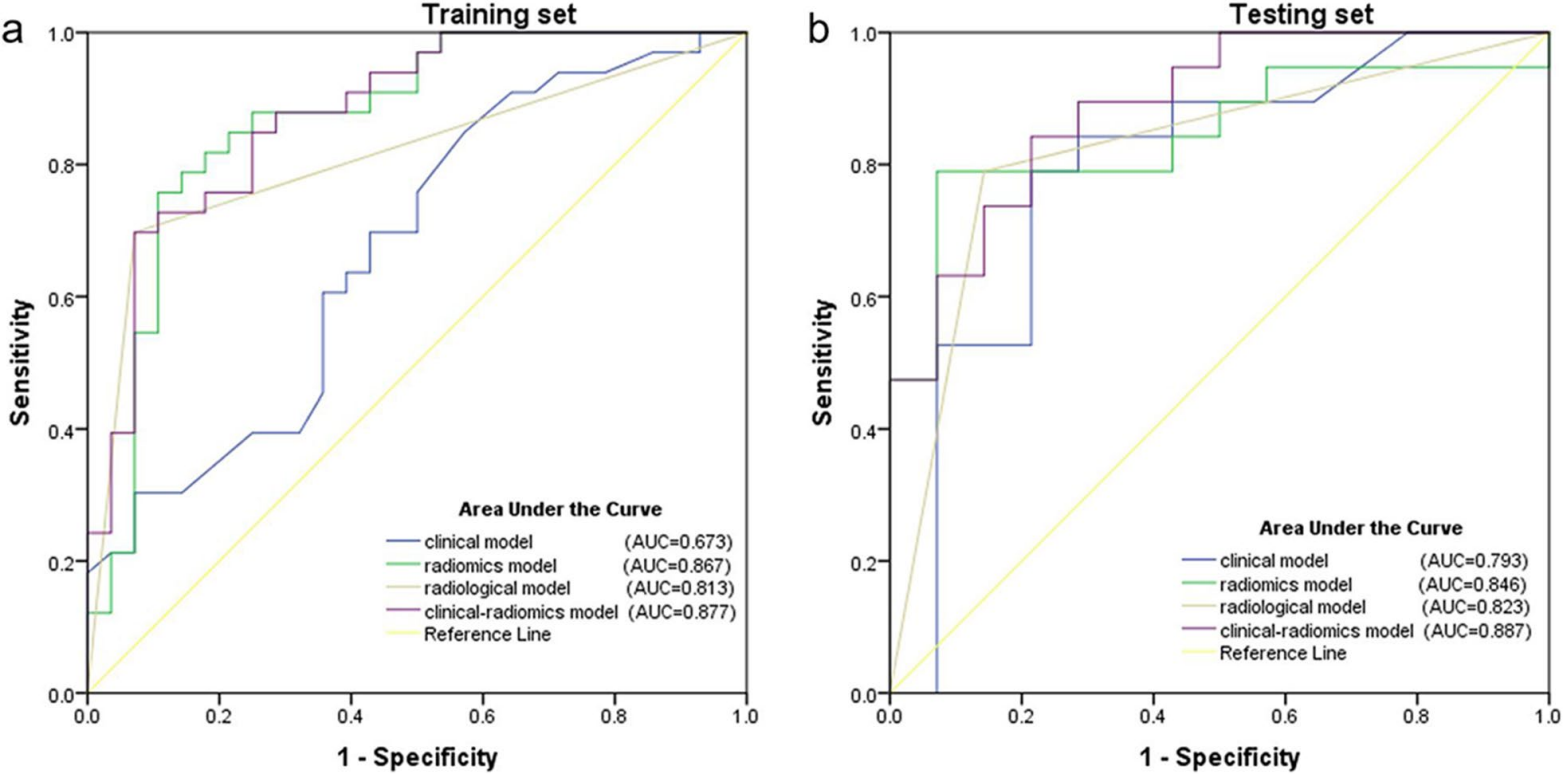
The ROC curves of the clinical (age and menopause), radiomics, radiological, and clinical–radiomics models for staging CC in the training (**a**) and the testing cohorts (**b**). The clinical–radiomics model yielded the highest AUCs for staging CC in the training cohort (AUC = 0.877) and the testing cohort (AUC = 0.887)

**Fig. 6:**
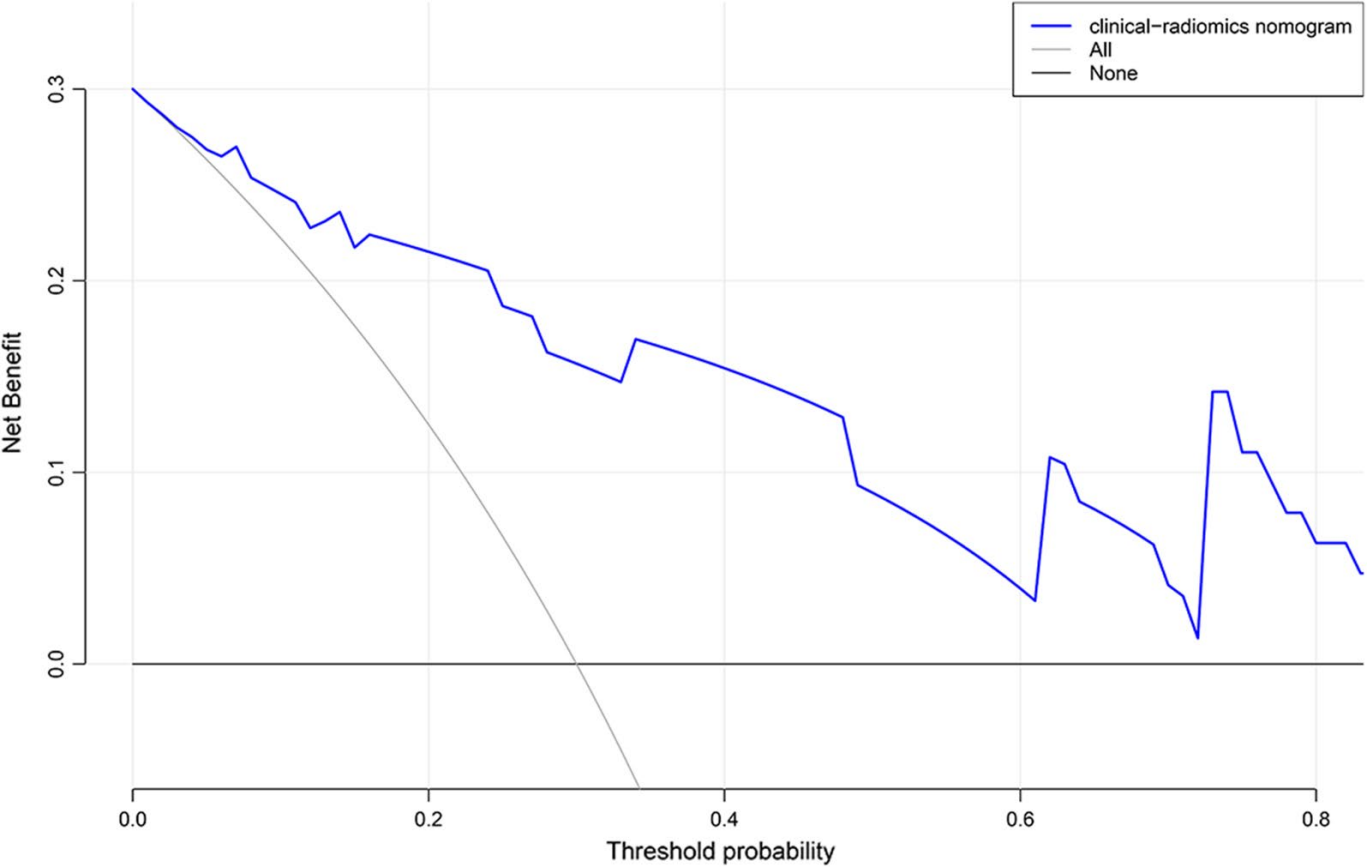
DCA of the clinical–radiomics model in the testing group. The decision curve demonstrates that the clinical–radiomics model enables obtaining more net benefits for making clinical decisions if the threshold is greater than 0.1

The clinical–radiomics nomogram was plotted based on clinical–radiomics model in the training set (Fig. 4). The predictive performance of the clinical, radiomics, radiological, and clinical–radiomics models for staging CC is shown in Table 5 and Fig. 5. In the training cohort, the clinical, radiomics, radiological, and clinical–radiomics models yielded AUCs of 0.673, 0.867, 0.813, and 0.877, respectively. For pairwise comparisons of the above models, the clinical–radiomics and radiomics models showed similar diagnostic performance, but significantly higher than that of the clinical model (*p* = 0.004 and 0.018, respectively). The differences of AUC between the remaining models were not statistically significant (all *p* > 0.05). In the testing cohort, the clinical–radiomics model (AUC = 0.887) yielded the highest predictive performance for staging CC when compared with the clinical model (AUC = 0.793, *p* = 0.095), radiomics model (AUC = 0.846, *p* = 0.433), and radiological model (AUC = 0.823, *p* = 0.400), but the differences were not statistically significant. The comparison of AUC between each model has been added in detail in Additional file 1: Table S2. DCA demonstrated that when the threshold probability was greater than 10%, the clinical–radiomics model could bring more net benefit than the treat-all or treat-none strategies (Fig. 6).

**Table 5:** Predictive performance of the clinical (age and menopause), radiomics, radiological, and clinical–radiomics models for delineating early stage from advanced-stage cervical cancer

	AUC	Sensitivity	Specificity	*p* value
*Training set*				
Clinical model	0.673 (0.540, 0.787)	0.849 (0.681, 0.949)	0.429 (0.245, 0.628)	0.004
Radiomics model	0.867 (0.755, 0.940)	0.758 (0.577, 0.889)	0.893 (0.718, 0.977)	0.551
Radiological model	0.813 (0.692, 0.901)	0.697 (0.513, 0.844)	0.929 (0.765, 0.991)	0.206
Clinical–radiomics model	0.877 (0.767, 0.947)	0.697 (0.513, 0.844)	0.929 (0.765, 0.991)	Ref
*Testing set*				
Clinical model	0.793 (0.617, 0.914)	0.790 (0.544, 0.939)	0.786 (0.492, 0.953)	0.095
Radiomics model	0.846 (0.678, 0.947)	0.790 (0.544, 0.939)	0.929 (0.661, 0.998)	0.433
Radiological model	0.823 (0.651, 0.933)	0.790 (0.544, 0.939)	0.857 (0.572, 0.982)	0.400
Clinical–radiomics model	0.887 (0.729, 0.970)	0.842 (0.604, 0.966)	0.786 (0.492, 0.953)	Ref

Parentheses indicate 95% confidence interval

*AUC* Area under the curve, *Ref* Reference

*p* value means the difference of the models compared with the Ref based on DeLong test

## Discussion

In our study, we developed and validated a model combining the rFOV DWI-derived radiomics with clinical features in predicting the stage of CC. The clinical–radiomics model yielded the highest predictive performance of staging CC when compared with the individual clinical model, radiomics model, and the radiological model. With these results, our study indicated the feasibility of clinical–radiomics model as a potentially noninvasive tool in staging CC, thereby aiding in the selection of therapeutic strategy [16].

During the past years, radiomics has been successfully recognized as a useful imaging tool in exploring the heterogeneity of many kinds of cancers [17–19], including cervical cancer [12, 14, 15, 20–23]. Liu et al. [14] evaluated that the whole-tumor volumetric 3D radiomics analysis had a better performance in stratifying the histological grade of CC. Deng et al. [24] proved that radiomics prediction models demonstrated the potential to noninvasively differentiate LNM and VEGF expression in CC. However, the previous study has conducted a model based on the radiomics features derived from the conventional DWI, which is susceptible to artifacts and distortion [22]. The ROIs derived from these conventional sequences may deviate from the real tumor range, which would result in the failure of the extracted radiomics features to truly reflect the heterogeneity of the tumors, thus affecting the discriminant performance of the model.

Unlike previous studies, the radiomics features were extracted from the rFOV DWI in this study. By employing two-dimensional space selective excitation pulses and 180° refocusing pulses, thereby reducing the number of baselines required for k-space filling in the phase direction, rFOV DWI is able to reduce the field of view, alleviate image artifacts, and thereby improve the image quality [25]. Compared with the conventional DWI, rFOV DWI could produce superior resolution and better identification of anatomic structures. The rFOV DWI has been widely applied to spinal cord, breast, thyroid, pancreas, prostate, uterus, and bladder [26, 27]. As rFOV DWI could provide less anatomic distortion and better image quality [8, 28], the radiomics features extracted from rFOV DWI were supposed to better reflect the heterogeneity of tumors. Texture-based features (neighboring gray tone difference matrix, gray-level co-occurrence matrix) could be used to describe the distribution of voxel signal intensities, which correlates with tumor heterogeneity [29]. Considering the varying degrees of structural complexity among different tumor stages [30], the textural features selected in this study were supposed to be helpful in exploring the intra-tumor heterogeneity of CC [31, 32]. The AUC of the radiomics signature was 0.867 (95% CI: 0.755, 0.940) in the training cohort and 0.846 (95% CI: 0.678, 0.947) in the testing cohort. This result indicated that the radiomics features could be independent predictors of CC staging.

Given that previous studies suggested the age and menopausal status of patients were also independent factors in predicting the stage of CC [33–35], these two clinical factors were incorporated into the clinical–radiomics model. Finally, the clinical–radiomics model containing the rad-score, age, and menopausal status was established to predict the stage in patients with cervical cancer. Most of the previous articles simply built a single radiomics model [22], and few built multiple models related clinical factors, radiomics features, and subjective evaluation of stage, as did in this study. In this study, we compared the predictive performance of the clinical, radiomics, radiological, and clinical–radiomics models for CC staging in the training and testing cohorts. Our results showed that clinical–radiomics model achieved the highest predictive performance with an AUC of 0.877 (95% CI, 0.767, 0.947) in the training cohort and 0.887 (95% CI, 0.729, 0.970) in the testing cohort. It indicated that the clinical–radiomics model may have advantages over the radiomics features or subjective assessment based on MRI for staging CC. With these results, our study demonstrated the feasibility of using the clinical–radiomics model as a potential noninvasive tool to complement staging of CC.

There are still some limitations in this study. This study was a retrospective study, which may lead to selection bias. The sample size was small and it was a single-center study, so a prospective multi-center study is needed to further verify our results. The delineation of VOI was only based on the sagittal ADC map, and other sequences were not combined. Therefore, the next step is to increase the sample size, conduct a multi-center study, and further improve the study by combining other MRI sequences.

In conclusion, we demonstrated that the clinical–radiomics model combining rFOV DWI-derived radiomics features and clinical indicators (age and menopausal status) may be of potential in staging CC, thereby providing more information for individualized treatment planning and prognosis evaluation in patients with CC.

## Supplementary Information

**Additional file 1**. Supplementary material.

## Data Availability

All the data are available from the corresponding author on reasonable request.

## Abbreviations

3D: Three-dimensional
ADC: Apparent diffusion coefficient
AUC: Area under the curve
CC: Cervical cancer
CI: Confidence interval
DCA: Decision curve analysis
DICOM: Digital imaging and communications in medicine
FIGO: International federation of gynecology and obstetrics
ICC: Intra-class correlation coefficient
LASSO: Least absolute shrinkage and selection operator
rFOV: Reduced field-of-view
ROC: Receiver‑operating characteristic
VOI: Volume of interest
