# Supplementary material for: Reduced field-of-view DWI‑derived clinical–radiomics model for the prediction of stage in cervical cancer
Source: Insights Imaging. 2023 Jan 26;14:18. doi: 10.1186/s13244-022-01346-w (PMC9880109; doi:10.1186/s13244-022-01346-w)
Supplement: Supplementary file 1 — Additional file 1. Supplementary material. [file 13244_2022_1346_MOESM1_ESM.pdf]

ELECTRONIC SUPPLEMENTARY MATERIAL

Reduced field-of-view DWI-derived clinical-radiomics model for the prediction of stage in cervical cancer

Appendix 1. Rad-score calculation formula:

Rad-score = -25.629043392 - 1.883115529 × Original_ngtdm_Contrast - 0.982822030 × Wavelet-LLH_glcm_InformationalMeasureofCorrelation2 + 32.062049922 × Wavelet-LHH_glcm_InverseDifferenceMomentNormalized - 0.001128832 × Wavelet-LLL_firstorder_RobustMeanAbsoluteDeviation - 0.812853027 × Wavelet-LLL_ glcm_ DifferenceEntropy

Table S1. Detailed information of radiomics features extracted from the ADC maps images.

Image type	Feature classes	Numbers of features	Definite features
original	shape	14	Elongation, Flatness, LeastAxisLength, MajorAxisLength, Maximum2DDiameterColumn, Maximum2DDiameterRow, Maximum2DDiameterSlice, Maximum3DDiameter, MeshVolume, MinorAxisLength, Sphericity, SurfaceArea, SurfaceVolumeRatio, VoxelVolume
original	firstorder	18	10Percentile, 90Percentile, Energy, Entropy, InterquartileRange, Kurtosis, Maximum, MeanAbsoluteDeviation, Mean, Median, Minimum, Range, RobustMeanAbsoluteDeviation, RootMeanSquared, Skewness, TotalEnergy, Uniformity, Variance
original	glcm	24	Autocorrelation, ClusterProminence, ClusterShade, ClusterTendency, Contrast, Correlation, DifferenceAverage, DifferenceEntropy, DifferenceVariance, Id, Idm, Idmn, Idn, Imc1, Imc2, InverseVariance, JointAverage, JointEnergy, JointEntropy, MCC, MaximumProbability, SumAverage, SumEntropy, SumSquares
original	gldm	14	DependenceEntropy, DependenceNonUniformity, DependenceNonUniformityNormalized, DependenceVariance, GrayLevelNonUniformity, GrayLevelVariance, HighGrayLevelEmphasis, LargeDependenceEmphasis, LargeDependenceHighGrayLevelEmphasis, LargeDependenceLowGrayLevelEmphasis, LowGrayLevelEmphasis, SmallDependenceEmphasis, SmallDependenceHighGrayLevelEmphasis, SmallDependenceLowGrayLevelEmphasis

original	glrlm	16	GrayLevelNonUniformity, GrayLevelNonUniformityNormalized, GrayLevelVariance, HighGrayLevelRunEmphasis, LongRunEmphasis, LongRunHighGrayLevelEmphasis, LongRunLowGrayLevelEmphasis, LowGrayLevelRunEmphasis, RunEntropy, RunLengthNonUniformity, RunLengthNonUniformityNormalized, RunPercentage, RunVariance, ShortRunEmphasis, ShortRunHighGrayLevelEmphasis, ShortRunLowGrayLevelEmphasis
original	glszm	16	GrayLevelNonUniformity, GrayLevelNonUniformityNormalized, GrayLevelVariance, HighGrayLevelZoneEmphasis, LargeAreaEmphasis, LargeAreaHighGrayLevelEmphasis, LargeAreaLowGrayLevelEmphasis, LowGrayLevelZoneEmphasis, SizeZoneNonUniformity, SizeZoneNonUniformityNormalized, SmallAreaEmphasis, SmallAreaHighGrayLevelEmphasis, SmallAreaLowGrayLevelEmphasis, ZoneEntropy, ZonePercentage, ZoneVariance
original	ngtdm	5	Busyness, Coarseness, Complexity, Contrast, Strength
wavelet-LLH	Firstorder, glcm, gldm, glrlm, glszm, ngtdm	18+24+14+16+16+5	Same as above
wavelet-LHL	Firstorder, glcm, gldm, glrlm, glszm, ngtdm	18+24+14+16+16+5	Same as above

wavelet-LHH	Firstorder, glcm, gldm, glrlm, glszm, ngtdm	18+24+14+16+16+5	Same as above
wavelet-HLL	Firstorder, glcm, gldm, glrlm, glszm, ngtdm	18+24+14+16+16+5	Same as above
wavelet-HLH	Firstorder, glcm, gldm, glrlm, glszm, ngtdm	18+24+14+16+16+5	Same as above
wavelet-HHL	Firstorder, glcm, gldm, glrlm, glszm, ngtdm	18+24+14+16+16+5	Same as above
wavelet-HHH	Firstorder, glcm, gldm, glrlm, glszm, ngtdm	18+24+14+16+16+5	Same as above
wavelet-LLL	Firstorder, glcm, gldm, glrlm, glszm, ngtdm	18+24+14+16+16+5	Same as above

NOTE: GLCM: Gray Level Co-occurrence Matrix. GLDM: Gray Level Dependence Matrix. GLRLM: Gray Level Run Length Matrix. GLSZM: Gray Level Size Zone Matrix. NGTDM: Neighbouring Gray Tone Difference Matrix.

Table S2. The pairwise comparison of ROC curves between each model based on DeLong test.

	P values for comparison
**Training set**	
Clinical model vs Radiomics model	0.018
Clinical model vs Radiological model	0.092
Clinical model vs Clinical-radiomics model	0.004
Radiomics model vs Radiological model	0.319
Radiomics model vs Clinical-radiomics model	0.551
Radiological model vs Clinical-radiomics model	0.206
**Testing set**	
Clinical model vs Radiomics model	0.574
Clinical model vs Radiological model	0.784
Clinical model vs Clinical-radiomics model	0.095
Radiomics model vs Radiological model	0.753
Radiomics model vs Clinical-radiomics model	0.433
Radiological model vs Clinical-radiomics model	0.400
